# Modulation of the high-order chromatin structure by Polycomb complexes

**DOI:** 10.3389/fcell.2022.1021658

**Published:** 2022-10-05

**Authors:** Yiran Guo, Gang Greg Wang

**Affiliations:** ^1^ Lineberger Comprehensive Cancer Center, University of North Carolina at Chapel Hill School of Medicine, Chapel Hill, NC, United States; ^2^ Department of Biochemistry and Biophysics, University of North Carolina at Chapel Hill School of Medicine, Chapel Hill, NC, United States; ^3^ Curriculum in Genetics and Molecular Biology, University of North Carolina at Chapel Hill, Chapel Hill, NC, United States; ^4^ Department of Pharmacology, University of North Carolina at Chapel Hill School of Medicine, Chapel Hill, NC, United States

**Keywords:** Polycomb repressive complex, Polycomb associated domain, chromatin looping, phase separation, cohesin, H3K27me3, CTCF

## Abstract

The multi-subunit Polycomb Repressive Complex (PRC) 1 and 2 act, either independently or synergistically, to maintain and enforce a repressive state of the target chromatin, thereby regulating the processes of cell lineage specification and organismal development. In recent years, deep sequencing-based and imaging-based technologies, especially those tailored for mapping three-dimensional (3D) chromatin organization and structure, have allowed a better understanding of the PRC complex-mediated long-range chromatin contacts and DNA looping. In this review, we review current advances as for how Polycomb complexes function to modulate and help define the high-order chromatin structure and topology, highlighting the multi-faceted roles of Polycomb proteins in gene and genome regulation.

## Introduction

**FIGURE 1:**
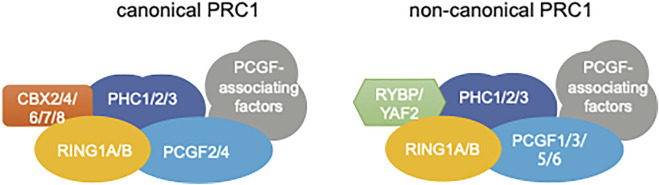
Subunit composition of Polycomb repressive complex 1 (PRC1), either canonical PRC1 (cPRC1, left) or non-canonical PRC1 (ncPRC1; right). The cPRC1 complex is composed of four core subunits, include a RING (either RING1A or RING1B), a PHC protein, a CBX (either CBX2, CBX4, CBX6, CBX7, or CBX8), a PCGF (either PCGF2 or PCGF4), in addition to associating proteins. For the ncPRC1 complex, CBX is replaced by either RYBP or YAF2, the PCGF being either PCGF1, PCGF3, PCGF5, or PCGF6, in addition to other ncPRC1-specific associating factors.

The lysine methyltransferase EZH2, together with EED, SUZ12 and RBBP4/7, forms the PRC2 “core” complex that is sufficient to conduct progressive methylation of histone H3 lysine 27 (H3K27) *in vitro* (Kuzmichev et al., 2002; Cao and Zhang, 2004; Margueron and Reinberg, 2011; Guo et al., 2021; Blackledge and Klose, 2021). An EZH2-related enzyme, EZH1, was reported to have similar cellular functions in PRC2 assembly and H3K27me3 deposition (Margueron et al., 2008; Shen et al., 2008). In cells, the PRC2 “core” complex additionally associates with a set of auxiliary cofactors, which can either facilitate the chromatin targeting or mediate functional modulation of PRC2. On the other hand, the PRC1 “core” complex is established by a RING1 catalytic subunit (either RING1A or related RING1B) (Wang et al., 2004), a PHC protein (PHC1, PHC2, or PHC3), a PCGF protein (PCGF1-6), and a CBX protein [CBX2/4/6/7/8; which contains a chromodomain that can recognize H3K27 trimethylation (H3K27me3)] (Blackledge and Klose, 2021; Kim and Kingston, 2022). The CBX subunit can be replaced by YAF2 or RYBP. Based on the composition of complex subunits, PRC1 can be divided into the canonical PRC1 (cPRC1, which contains a CBX) or non-canonical PRC1 (ncPRC1, which contains either YAF2 or RYBP but not CBX) (Figure 1). PRC2 and PRC1 serve as the primary “writer” enzyme for H3K27me3 and mono-ubiquitination of histone H2A Lys119 (H2AK119ub), respectively. PRC2 and PRC1 can act in concert or independently to maintain a repressive state at target chromatin regions. Part of PRCs-associated repressive activities can be attributed to PRC’s enzymatic product *per se* (i.e., H3K27me3 and H2AK119ub). For example, somatic heterozygous gain-of-function mutation of EZH2 at its residue Tyr-646 occurs frequently in human B cell lymphoma patients, which leads to a global increase of H3K27me3 and a more closed chromatin structure (Sneeringer et al., 2010; Xu et al., 2015; Donaldson-Collier et al., 2019). PRC complex-generated histone modifications, H3K27me3 and H2AK119ub, can serve as a docking site for recruiting “reader” or effector proteins, which subsequently mediate the processes of gene silencing and/or chromatin compaction. Formation of various PRC1/2 sub-complexes and their respective gene-regulatory functions have been extensively reviewed elsewhere and readers shall refer to recent comprehensive review articles (Margueron and Reinberg, 2011; Vizan et al., 2015; Schuettengruber et al., 2017; Deevy and Bracken, 2019; Blackledge and Klose, 2021; Guo et al., 2021; Piunti and Shilatifard, 2021; Kim and Kingston, 2022; Parreno et al., 2022).

Increasing evidence points to critical roles of three-dimensional (3D) chromatin organization and high-order structure during organismal development and cell differentiation, and deregulation of chromatin topology is associated with development of human diseases (Bonev and Cavalli, 2016; Yu and Ren, 2017; Hansen et al., 2018; Hug and Vaquerizas, 2018; Brien and Bracken, 2019; Zheng and Xie, 2019; Quiroga et al., 2022). In recent years, deep sequencing- and imaging-based technologies developed for mapping 3D chromatin structure have shed new light on spatiotemporal organization of 3D chromatin organization and long-range contacts. Increasing evidence also shows that high-order chromatin structure is established at multi-level genomic length scales and can be regulated by a variety of molecular mechanisms.

**FIGURE 2:**
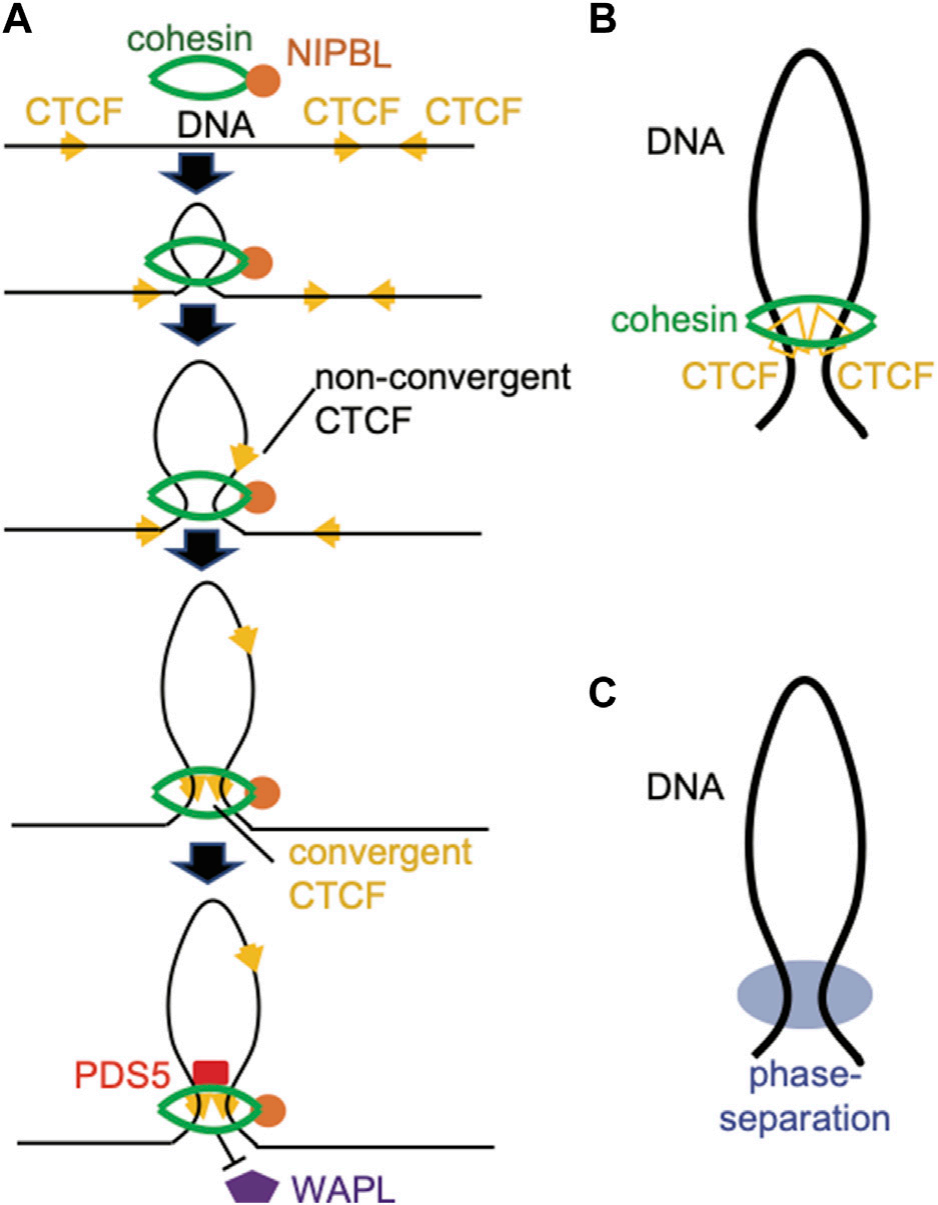
Schematic of 3D genome structure. **(A)** Illustration of a loop extrusion model, in which NIPBL, stabilized by BRD4, loads the ring-like cohesin complex onto target chromatin and activates its ATPase to extrude the DNA fiber. A pair of convergent CTCF proteins, and not those in the non-convergent configuration, stall cohesin’s sliding and prevent WAPL from releasing the cohesion complex off chromatin. PDS5 binds cohesin to stabilize its residence on the loop anchor. **(B)** Long-range chromatin interactions driven by the cohesion complex, with CTCFs serving as anchors. **(C)** Long-range chromatin contacts driven by a phase separation-based mechanism.

First, segregating chromatin regions can be achieved through DNA loops formed in the interphase cells. Here, the “loop extrusion” model (Figures 2A,B) is the most prevalent one to explain the formation of chromatin looping. In this model, cohesin complex, a multi-subunit ring-like motor protein machinery, uses the ATP hydrolysis-generated energy to extrude DNA and progressively enlarge DNA loops until it is stalled by the CCCTC-binding factor (CTCF) (Sanborn et al., 2015; Fudenberg et al., 2016; Merkenschlager and Nora, 2016; Wutz et al., 2017; Rowley and Corces, 2018; Davidson and Peters, 2021; Kraft et al., 2022) (Figure 2A). Further studies demonstrated that cohesin complex is loaded to DNA through the Nipped-B-like (NIPBL) protein and its binding partner MAU2, which are strong activators of cohesin’s ATPase activity (Davidson et al., 2019; Kim et al., 2019). In addition, BRD4, a histone acetylation “reader” and general transcriptional co-activator, recruits NIPBL and/or stabilizes its binding (Olley et al., 2018; Luna-Pelaez et al., 2019; Linares-Saldana et al., 2021). Cohesin can be either stabilized by PDS5 or released by WAPL (Vaur et al., 2012; Tedeschi et al., 2013) (Figure 2A). Loop extrusion by cohesin is terminated until it is anchored by a pair of CTCFs in a convergent direction (Rao et al., 2014). Such a convergent CTCF rule can be explained by a fact that an N-terminal region of CTCF (amino acids 222–231 of human CTCF) binds the SA2-SCC1 subunit of cohesin, thereby prohibiting WAPL binding to sustain cohesin on the chromatin (Li et al., 2020) (Figures 2A,B).

Loops are further folded into the self-interacting genomic regions, often termed Topologically Associating Domains (TADs), which span hundreds of kilobase pairs (Kbp) of DNA with boundaries usually anchored by CTCF. Chromatin in the same TADs shares similar chromatin state and transcriptional activity and is interactive and generally avoid of interactions to other domains, thereby regulating the transcription of embedded genes in a coordinated fashion (Rao et al., 2014).

At a larger genome scale (∼1 Mbp or more), chromatin forms two distinctive compartments, referred to as the A (active) and B (inactive) compartment, which display an enhanced frequency of contacts among themselves and correspond to open (A-type) and closed/compact (B-type) chromatin, respectively (Lieberman-Aiden et al., 2009).

Recent studies lend strong support for a notion that PRC complexes have important functions in spatial determination and fine-tuning of high-order chromatin structure. In this review, we discuss about recent advances on these topics.

## Polycomb repressive complexes mediate CCCTC-binding factor-independent chromatin contacts in *Drosophila*

By generating a sub-kilobase-resolution (500bp) Hi-C map in the cultured Kc167 *Drosophila melanogaster* embryonic cells, Eagen et al. (2017) found that over 70% of chromatin loops exhibit a general lack of CTCF binding at their anchors, which is in stark contrast to a high percentage (about 86%) of loop anchors actually associated with CTCF in human cells (Figures 2A,B). A large majority (72.8%) of chromatin loop anchors in *Drosophila* cells, however, did display binding by Rad21, a subunit of cohesin complex, indicating a possibly distinct loop formation mechanism mediated by cohesin in the fly (Eagen et al., 2017). In addition, a significant fraction of loop anchors in fly cells was found associated with binding of Polycomb complex—in fact, approximately a quarter of all chromatin loop have Polycomb binding (PRC1 and H3K27me3) at both anchors (Eagen et al., 2017). Of note, the Polycomb-associated chromatin loops in *Drosophila* tend to target the genes related to transcription regulation and development, and the promoters associated with Polycomb-bound loop anchors exhibit significantly lower activities in transcription than those without association to Polycomb loop anchors, suggesting a repressive function of these PRC-bound chromatin loops (Eagen et al., 2017).

Separate studies further revealed that both PRC1 and PRC1-associated DNA motifs (Polycomb Response Elements or PREs) are necessary for stabilization of chromosome architecture and maintenance of gene repression in the fly (Cheutin and Cavalli, 2018; Ogiyama et al., 2018). Using DNA-FISH and RNA-FISH technologies, Cheutin and Cavalli focused on the roles of Ph (homologous to PHC proteins in the mammalian PRC1 complex; Figure 1) and Pc (homologous to mammalian CBX proteins; Figure 1) in the formation of PRC1 chromatin loops at HOX loci during *Drosophila* embryogenesis and found that PRC1-associated chromatin folding imposes an architectural effect leading to gene repression (Cheutin and Cavalli, 2018). This work also illustrated that the loss of PRC1-associated loops proceeds gene derepression, indicating a causal effect of looping (Cheutin and Cavalli, 2018). Also, Ph and Pc show rather distinct functions in this biological process—loss of Ph led to diffusion of nuclear Pc and more significant gene derepression in fly embryos; in contrast, Ph proteins still accumulated as the nuclear foci in those Pc-null embryos and Pc-loss-associated gene derepression was found much weaker (Cheutin and Cavalli, 2018). Similar to what was observed with deletion of Polycomb proteins (Ph and Pc), perturbation of PRE also disrupted the 3D chromatin architecture at target regions (Ogiyama et al., 2018). As discussed above, the PRC-mediating DNA loops in *Drosophila* appear independent of CTCF; however, involvement of cohesin complex in PRC loop formation remains to be fully understood.

## In mammals, polycomb complexes are involved in long-range interaction between the poised enhancer/silencer and promoter for dynamic transcription regulation

In mammalian cells, Hi-C or Hi-ChIP methods uncovered about ten thousands of DNA loops and 4,101 loops were discovered by Hi-ChIP for H3K27me3 in mouse embryonic stem cells (mESCs) (Mumbach et al., 2016; Roayaei Ardakany et al., 2020; Kraft et al., 2022). PRC complexes also regulate the 3D chromatin structure in the mammals (Denholtz et al., 2013; Schoenfelder et al., 2015; Wani et al., 2016; Fursova et al., 2019). During transition from the native human pluripotent stem cells to primed ones, which mimics the pre- to post-implantation stages of embryonic development, acquisition of H3K27me3 is concomitant with the emergence of Polycomb-mediated long-range interactions (Chovanec et al., 2021). In mESCs, key developmental genes, such as Hox gene clusters, are embedded within Polycomb bodies formed partly by PRC1 and partners (Schoenfelder et al., 2015). Depletion of Ring1A/B disrupted the spatial interactions among Hox promoters (Schoenfelder et al., 2015). In agreement with this observation, the nuclear clustering pattern of PRCs’ target loci was found disrupted following the depletion of RING1B in mESCs, which points to a PRC1-dependent chromatin compaction (Boyle et al., 2020). Importantly, such an effect by PRC1 is independent of its intrinsic enzymatic activity since a RING1B catalytically-dead mutation (RING1BI53A) did not affect PRC1-dependent compaction (Boyle et al., 2020). Correlating gene expression with RING1B’s binding further revealed that, in the absence of PRC1, genes with RING1B binding at proximal sites (0–50 kb) were found upregulated whereas those with distal RING1B peaks (>100 kb) were not, indicating that PRC1’s repressive effect in mESCs is mainly proximal and not at a larger genomic scale (Boyle et al., 2020).

### Polycomb repressive complex maintain interactions between the poised enhancers and promoters

Long-range looping interaction between promoter and enhancer represents a common means for modulating transcription. Enhancers are distal gene-regulatory elements, which contain a characteristic mono-methylation of histone H3 lysine 4 (H3K4me1) (Creyghton et al., 2010; Rada-Iglesias et al., 2011). In addition to H3K4me1, the poised enhancers (PEs) are characterized by an additional presence of H3K27me3 and a lack of H3K27 acetylation (H3K27ac), while those active enhancers are marked by the presence of H3K27ac and a lack of H3K27me3; additionally, a class of so-called primed enhancers carry H3K4me1 with no modification of H3K27 (neither H3K27ac nor H3K27me3), which was viewed to be an intermediate stage between being repressive/poised and being fully activated (Creyghton et al., 2010; Rada-Iglesias et al., 2011).

Previously, it has been reported that PEs establish physical interactions with target genes in mESCs and even prior to cell differentiation (Bonev et al., 2017). In this scenario, PEs often display association with those developmentally critical genes, notably ones involved in body patterning and early development (Schoenfelder et al., 2015; Bonev et al., 2017; Cruz-Molina et al., 2017; Crispatzu et al., 2021). It was proposed that the PRC maintains a repressive state *via* PE-promoter contact in undifferentiated stem cells, which then enables a quick response to differentiation-stimulating signals (for example, gene activation upon loss of PRC) (Gentile et al., 2019). In agreement, the H3K27me3-decorated chromatin regions are often partially overlapped with active histone marks and located at the active chromatin compartments, although PRC2 target regions are generally more condensed (Vieux-Rochas et al., 2015; Mas et al., 2018; Xu et al., 2018). Also, regulation of enhancer-promoter (E-P) interaction seems to rely more on PRC1 and not H3K27me3, since the retention of H3K27me3 is not sufficient to maintain E-P interaction upon neuron differentiation (Bonev et al., 2017). However, PRC2 loss in differentiating limbs severely compromised the transcriptional induction of E-P-associated targets, as its depletion disrupts the interaction between HoxA genes and remote enhancers (Gentile et al., 2019). Further investigation is merited to dissect exact roles of PRC1 versus PRC2, as well as their respective enzymatic products (H3K27me3 and H2AK119ub), in regulation of E-P interactions and target gene expression during the mammalian development.

### Polycomb repressive complex bring the “silencer” elements to proximity for repressing gene expression

Like enhancers, silencers are distal cis-regulatory regions but usually harbor the repressive histone marks. Silencers can mediate gene repression when being brought to promoters *via* DNA looping; meanwhile, they can turn into enhancers under different contexts (Doni Jayavelu et al., 2020; Pang and Snyder, 2020; Segert et al., 2021). Recently, Ngan et al. (2020) reported that PRC2 mediates extensive looping between promoters, or between promoters and distal intergenic or intragenic enhancers, in mESCs; further, they showed that a subset of PRC2-enforced chromatin loops physically connect distal regulatory elements (DREs) with development-related genes, whose expression is known to be epigenetically silenced or poised in the undifferentiated mESCs. Disruption of PRC2-bound DRE caused the loss of PRC2-associated chromatin loop, decrease of H3K27me3, and de-repression of the target gene (Oksuz et al., 2018; Ngan et al., 2020). Thus, akin to how enhancers activate target gene expression *via* (co)activator-associated chromatin looping, these PRC2-bound DREs function as “silencers” and repress target gene transcription through chromatin looping associated with PRC2 and (co)repressors (Ngan et al., 2020). Not surprisingly, these “silencers” can turn into active enhancers during the development and lineage specification, demonstrating a dynamic nature of DREs (Ngan et al., 2020). These findings are in agreement with what was reported in a separate study showing that, in hematological cancer cell lines, the H3K27me3-rich genomic regions interact preferentially with each other, some of which serve as “silencers” for regulating gene expression *via* chromatin looping (Cai et al., 2021).

## Polycomb repressive complex-mediated and CCCTC-binding factor-mediated chromatin looping mechanisms operate independently, pointing to a multi-level regulation of 3D chromatin structure

### Polycomb associated domains

Polycomb complexes have been shown to mediate the formation of self-interacting domains of compacted chromatin, sometimes referred to as Polycomb Associated Domains or PRC1-associated domains (PADs), in animal cells (Denholtz et al., 2013; Schoenfelder et al., 2015; Kundu et al., 2017; Ogiyama et al., 2018; Boyle et al., 2020; Du et al., 2020). Similar PADs or PRC-repressed compartments were reported in the plant as well (Huang et al., 2021). Like TADs, chromatin inside the same PAD is self-interacting, which is essential for the maintenance of appropriate gene and chromatin state. For instance, during the neuronal differentiation of mESCs, those PADs harboring neuron-specific genes were found deconstructed (Bonev et al., 2017; Kundu et al., 2017). Interestingly, the PAD deconstruction was found correlated with a decrease of RING1B targeting and not H3K27me3, suggesting the latter histone mark is not necessary for PAD maintenance (Bonev et al., 2017). In early embryo, Polycomb complexes also drive dynamic loop formation and some of these loops could be important for gene imprinting (Chen and Zhang, 2020; Du et al., 2020). Disruption of PRC1, but not PRC2, attenuated PADs in the fully grown oocytes, although the loop anchors are covered by H3K27me3 (Du et al., 2020). Though H3K27me3 appears neither sufficient nor necessary to maintain PADs, it is required for the re-establishment of PADs in the two-cell stage of early mouse embryos (Du et al., 2020). Collectively, these studies demonstrated that Polycomb complexes, especially cPRC1, induce loop formation between DREs and genes involved in early development; and PRC2 may play a role in loop establishment, possibly by deposition of H3K27me3 to recruit cPRC1 at the nucleation sites, which could function as PADs’ anchors (Kraft et al., 2022).

### Polycomb associated domains or PRC1-associated domains are distinct from topologically associating domains

PADs are also distinct from TADs in terms of their regulatory factors and anchor features. First, unlike TADs, which are highly conserved in different tissues and even species, PADs could be highly dynamic (Du et al., 2020; Loubiere et al., 2020). Second, similar to what was seen in the fly cells, PADs in mESCs operate independently of CTCF since PADs are not anchored by CTCF and their formation was not altered without CTCF (Boyle et al., 2020). In contrast, TADs are often anchored by CTCF (Nora et al., 2017; Hansen et al., 2018). This indicates a multilevel regulation of 3D chromatin organization by different mechanisms such as the CTCF-driven one and PADs. However, previous studies of other biological processes also showed that CTCF often colocalizes with H3K27me3 marks and that CTCF can recruit PRC2 *via* interaction with SUZ12 to repress target genes (Li et al., 2008; Wang et al., 2020); and whether or not such a CTCF:PRC2 interaction regulates looping remains to be investigated.

Moreover, PADs are independent or even antagonized by cohesin complex, as PADs are not diminished and even enhanced upon cohesin removal (Du et al., 2020; Rhodes et al., 2020; Kriz et al., 2021). For example, depletion of SCC, a cohesin complex subunit, seems to enhance the chromatin interactions within PADs, indicating an antagonism between the two (Du et al., 2020). In agreement, the pro-longed cohesin residence inhibits the Xist-associated polycomb spreading and gene silencing, while removal of cohesin on active X chromosome causes the formation of mega-domain that is typically seen on inactivated X chromosome (Kriz et al., 2021). Interestingly, the above antagonism may also be cell type-specific, since it was observed only in mESCs and not in some examined cancer cells (Rhodes et al., 2020). A recent work further specified the role for different cohesin complex variants during the formation of PADs and found that cohesin-SA1 (STAG1) variant primarily exists on TADs’ boundaries whereas cohesin-SA2 (STAG2) variant colocalizes with PRC1 and facilitates Polycomb domain compaction through PRC1 recruitment (Cuadrado et al., 2019). Consistently, SA2 occupies PRC2-bound regulatory region in Ewing’s sarcoma cells and its binding is critical for gene regulation (Adane et al., 2021).

### Phase separation as a mechanism underlying polycomb repressive complex-mediated chromatin compaction

Phase separation such as liquid-liquid phase separation (LLPS) was proposed to be crucial for transcriptional control and chromatin structure organization (Hnisz et al., 2017). Ahn et al. (2021) have demonstrated that NUP98-HOXA9, an oncogenic transcription factor (TF) with potent LLPS capability, mediates CTCF-independent DNA loops among cognate NUP98-HOXA9-bound sites, forming long-range E:P contacts, and that such a looping mechanism requires the presence of NUP98-HOXA9’s intrinsically disordered regions (IDRs), which is essential for LLPS; similar looping was also reported for another cancer-related TF, EWS-FLI (Showpnil et al., 2022). Moreover, a recent work indicated that LLPS plays a role in weakening the insulation properties of a subset (∼20%) of TAD boundaries, which exhibit the feature of “house-keeping” genes, high transcription and a lack of CTCF (Gamliel et al., 2022). Mounting evidence points to critical roles for nuclear condensation and LLPS of TFs and chromatin factors, including PRC complexes, during the long-distance association and 3D organization of chromatin (Figure 2C).

**FIGURE 3:**
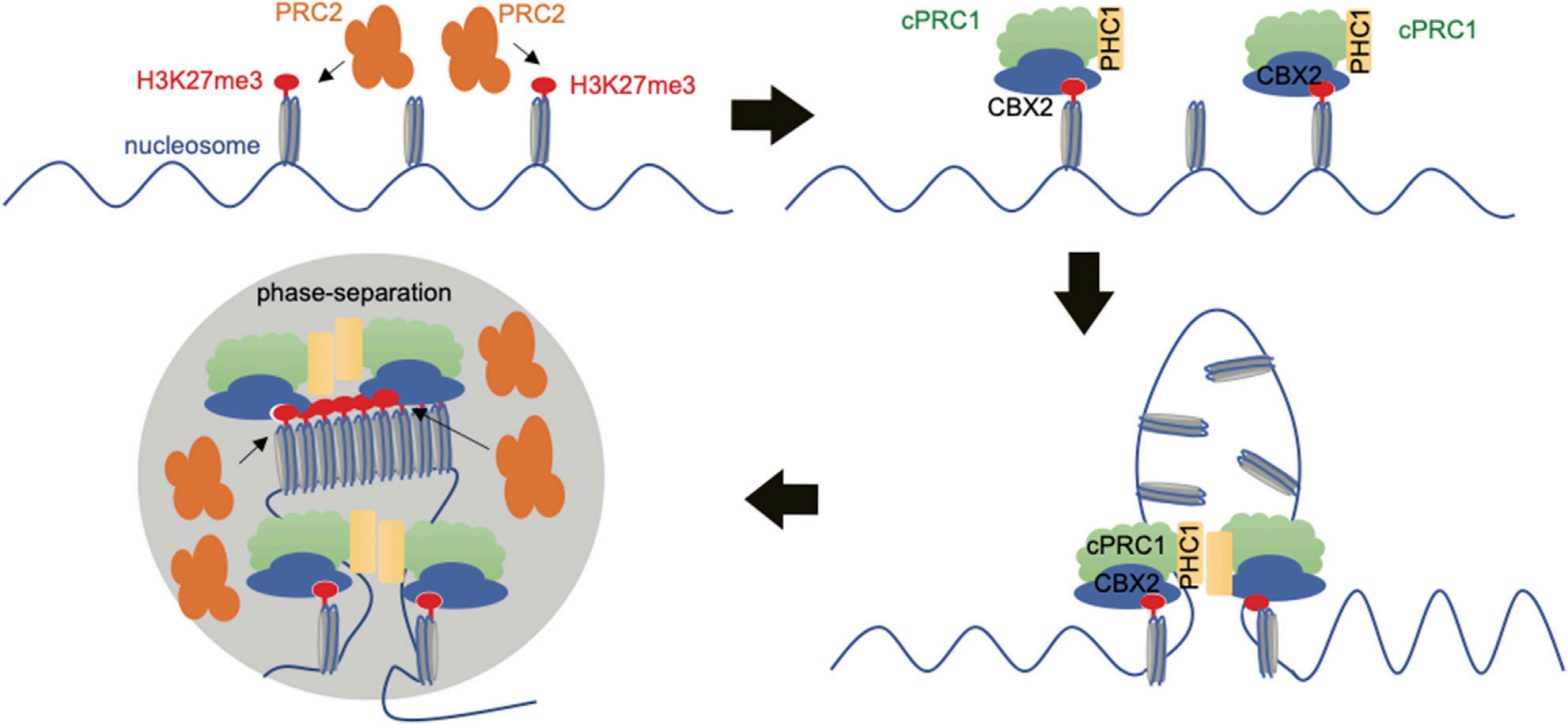
Schematic to show formation of Polycomb Associated Domains (PADs). PRC2 deposits H3K27me3, preferentially at regions with unmethylated CpG-rich sequence. H3K27me3 is then “read” by the CBX subunit of cPRC1. The cPRC1 variant complex then drives chromatin compaction by liquid-liquid phase separation (LLPS), which is at least achieved through the subunit of CBX2 (with its chromodomain reading H3K27me3 while its IDRs mediating LLPS) and/or PHC1 (with the SAM domain mediating LLPS). PRC2 deposits more H3K27me3 marks to maintain PADs and repression of the embedded genes.

Indeed, studies revealed that cPRC1 can form condensates through LLPS, which is driven by IDRs harbored within cPRC1’s subunits, CBX2 and PHC1, and that PRC1’s condensation and LLPS contribute to chromatin compaction and target gene silencing (Isono et al., 2013; Lau et al., 2017; Plys et al., 2019; Tatavosian et al., 2019; Seif et al., 2020; Eeftens et al., 2021). Here, LLPS may mediate chromatin contacts by locally concentrating PRC proteins and PRC-bound chromatin, thereby facilitating the spread of repressive histone marks (Cheutin and Cavalli, 2019; Guo et al., 2021) (Figure 3). In accordance with this notion, single-molecule tracking studies in live cells found that about 10% of PRC1, mostly cPRC1, stably binds chromatin at “Polycomb body,” which is a known phase-separated entity, in a H3K27me3-dependent manner (Huseyin and Klose, 2021). In addition, it has been shown that PRC2 and PRC1 deposit H3K27me3 and H2AK119ub, respectively, which further recruits PRC1/2 variant complex onto same chromatin regions (Blackledge and Klose, 2021; Guo et al., 2021; Kim and Kingston, 2022); thus, it is possible that a feedforward loop exists, promoting PRCs’ LLPS and PAD formation (Figure 3). Also, it is possible that CBX2 can nucleate on chromatin independently of H3K27me3 and other PRC1 subunits (Kent et al., 2020). Upon nucleation, CBX-PRC1 assembles condensates, and the consequence of this event is to shorten diffusion time in the 3D space and to reduce trials for finding specific sites through revisiting the same or adjacent sites repetitively, thereby accelerating CBX2-mediated searching for H3K27me3-decorated sites (Kent et al., 2020).

## Conclusion and outstanding questions

It becomes increasingly clear that PRC complexes play crucial roles in the determination and/or modulation of 3D chromatin structure, thereby regulating gene expression patterns during organismal development. In future, effort shall be made to further decipher causal roles for the recruitment and binding of PRC1, PRC2, loop anchors and various cofactors in the formation and maintenance of loops. Many of the works covered in this review used either early embryo or ESCs as model system and further research is warranted to investigate the dynamics of PRCs-mediated 3D chromatin organization during development, given that PRC components are dynamically expressed among various differentiated cells and tissue types. Furthermore, a phase separation model for PRC-mediated chromatin compaction (Figure 3) is appealing but requires further examination. In ESCs where CBX7 is the main CBX form in cPRC1, there are only about 10 PRC1 molecules per Polycomb body, which is lower than what would be required to support and establish the PRC1 phase separation (Huseyin and Klose, 2021). Thus, it needs further investigation whether or not LLPS underlies PRC-associated chromatin compaction under different biological contexts. Moreover, certain long-coding RNAs are critical for the genomic targeting of PRC2 complex to establish long H3K27me3 domains (Colognori et al., 2019; Schertzer et al., 2019; Trotman et al., 2021); here, it is possible that RNA and transcription can coordinate the 3D organization of chromatin thereby mediating PRC’s targeting and spreading. The detailed mechanism remains to be fully understood. In addition to the above discussed gene-repressive roles, PRC1 and some polycomb proteins such as EZH2 and RING1B were reported to have gene-activation functions as well (Mousavi et al., 2012; Cohen et al., 2018; Loubiere et al., 2020; Wang and Wang, 2020; Zhang et al., 2020; Zhang et al., 2021; Wang et al., 2022). Some of these gene-activation activities of polycomb proteins were suggested to be related to PRCs’ looping effect (Loubiere et al., 2020), others due to the intrinsic transactivation activity harbored within polycomb proteins such as EZH2 (Jiao et al., 2020; Wang et al., 2022), and the rest remain completely unknown, which merits additional studies. Lastly, Polycomb mis-regulation often perturbs the normal processes of development and cell differentiation, leading to pathogenesis. A better understanding of PRC-mediated gene and genome regulation shall aid in therapeutic development.
